# Global Research Trends in Pediatric Acute Respiratory Distress Syndrome: A Bibliometric Analysis from 2014 To 2024

**DOI:** 10.1007/s44197-025-00434-6

**Published:** 2025-06-20

**Authors:** Keke Chen, Chengjie Chen, Xiang Zheng, Sihu Chen, Guoquan Pan, Yafeng Liang

**Affiliations:** 1 Pediatric Emergency department, The second Affiliated Hospital, Yuying Children’s Hospital of Wenzhou Medical University, Wenzhou, 325000 China; 2 Pediatric Intensive Care Unit, The second Affiliated Hospital and Yuying Children’s Hospital of Wenzhou Medical University, Wenzhou, 325000 China; 3 Zhejiang Provincial Clinical Research Center for Pediatric Precision Medicine, Zhejiang, China

**Keywords:** Pediatric ARDS, Bibliometric analysis, ARDS, Respiratory failure

## Abstract

**Background:**

To assess an overview of research trends, influential studies, and collaborative networks in pediatric acute respiratory distress syndrome (PARDS), identifying key contributions and insights for future research directions.

**Methods:**

Web of Science Core Collection (WoSCC) database was used to conduct the bibliometric analysis. Bibliometric indicators such as publication counts, citation frequencies, authors, countries/regions, institutions and keyword occurrences were analyzed using VOSviewer and CiteSpace and R.

**Results:**

The analysis included 419 publications from 2014 to 2024, with a total of 88,849 citations. These publications involved 22,141 authors affiliated with various institutions worldwide. the United States leading in research output, contributing 1,124 articles and 24,421 citations. Leading institutions included the University of Pennsylvania, the University of California System, and the Children’s Hospital of Philadelphia. The most influential journals were *Pediatric Critical Care Medicine* and *Critical Care Medicine*. Yehya N, Thomas NJ, and Khemani RG are the most contributed authors. Keyword co-occurrence analysis revealed five major research clusters: neonatal outcomes, critical care strategies (e.g., mechanical ventilation and extracorporeal membrane oxygenation [ECMO]), inflammation and immune mechanisms, respiratory support interventions, and diagnostic tools like lung ultrasound. Emerging trends focused on inflammation, molecular mechanisms, and diagnostic advancements.

**Conclusion:**

This study presents a bibliometric analysis of PARDS research, highlighting significant trends, influential studies, and collaborative networks. The findings provide valuable insights into the current state of research and future directions for advancements, emphasizing the need for continued investigation to improve clinical outcomes for children with ARDS.

**Clinical Trial Number:**

Not applicable.

**Supplementary Information:**

The online version contains supplementary material available at 10.1007/s44197-025-00434-6.

## Background

Acute respiratory distress syndrome (ARDS) is a severe lung condition characterized by widespread inflammation, frequently leading to respiratory failure [1]. This syndrome poses notable challenges in pediatric patients due to their distinct physiological features and varied causes compared to adults. ARDS is a significant contributor to respiratory failure in children admitted to pediatric intensive care units (PICUs) [2], accounting for 1–10% of such admissions [3]. Mortality rates in pediatric ARDS (PARDS) differ across studies, influenced by diverse comorbidities and etiologies [3]. The main goal of ARDS treatments is to enhance oxygenation and improve patient outcomes, primarily by reducing mortality and morbidity. Reducing the healthcare burden is an important secondary objective [4]. However, managing ARDS in children demands tailored strategies that consider their developmental stages and disease manifestations [5]. The immature immune system and smaller airways in these patients necessitate cautious ventilation and fluid management to minimize lung damage [6]. Treatment decisions must also balance potential benefits with the risks of long-term pulmonary and neurodevelopmental effects [7].

PARDS often arises from severe infections, such as pneumonia and sepsis, which are among the leading causes of respiratory failure in children [8]. Traumatic injuries, including blunt trauma and near-drowning accidents, are also significant contributors to the development of PARDS [9]. In addition, children may develop ARDS secondary to underlying conditions like congenital heart disease or complications arising from medical interventions such as bone marrow transplantation [10]. For example, bone marrow transplantation can lead to pulmonary complications, including ARDS, due to the high risk of infection and immune responses associated with the procedure [11].

The global incidence of PARDS is significant and continues to grow. Studies report that it affects 2–12.8 per 100,000 children annually, with an incidence of 1–10% among those admitted to pediatric intensive care units (PICUs) [4, 9]. Despite the increasing attention and research on the condition, a comprehensive analysis that aggregates and synthesizes all available studies on PARDS is still lacking. This gap in the literature calls for a systematic bibliometric analysis to consolidate current knowledge, identify key research trends, and highlight areas requiring further investigation [12]. Such an analysis is essential for understanding the evolving research landscape of PARDS, including treatment methods, clinical outcomes, and the challenges faced in managing this condition [10]. By examining the existing body of literature, a bibliometric analysis can provide insights into research gaps, guide future studies, and inform clinical practice. Ultimately, this comprehensive approach is vital for improving clinical outcomes and developing more effective treatments for children with ARDS.

Bibliometric analyses have emerged as powerful tools in exploring the research landscape across various medical fields. These analyses offer deep insights into publication patterns, influential authors, and pivotal topics, thereby serving as invaluable resources for understanding the impact and progression of scientific literature within specialized areas [12, 13]. In the domain of PARDS, bibliometric studies have particularly aided in identifying key research trends and hotspots, such as the exploration of novel therapies and the examination of epidemiological factors [14, 15]. Bibliometric analyses have widely applied across various pediatric-related medical fields—including oncology, neurology, and infectious diseases—to provide critical insights into research output, impact, and collaborative networks [4, 16]. Despite the significant burden that ARDS poses on the pediatric populations, a comprehensive bibliometric study dedicated to PARDS is notably absent. Such a study would be pivotal in uncovering the research landscape, identifying key players and topics, and ultimately guiding future investigations aimed at improving clinical outcomes for children affected by this severe respiratory condition [7].

This bibliometric analysis is the first in PARDS area, aims to systematically identify and analyze research publications related to this condition. By synthesizing data from scholarly databases, this study seeks to elucidate the current state of research, highlight emerging trends, and identify gaps in knowledge, and guide future investigations and inform clinical practice, thereby enhancing outcomes for PARDS patients.

## Methods

### Search Strategies and Data Collection

We conducted a literature search using the Web of Science Core Collection (WoSCC) database to identify relevant publications on PARDS and acute lung injury (ALI). The search query was formulated as follows: (TS=(pediatric* OR youth OR baby OR babies OR infant* OR newborn* OR toddler* OR adolescent*)) AND TS=(ALI OR acute lung injury OR ARDS OR acute respiratory distress syndrome OR respiratory distress syndrome). The search was performed on October 16, 2024, with the time frame restricted to articles published from January 1, 2014, to October 16, 2024, to ensure data consistency and minimize discrepancies due to subsequent database updates.

**Fig. 1:**
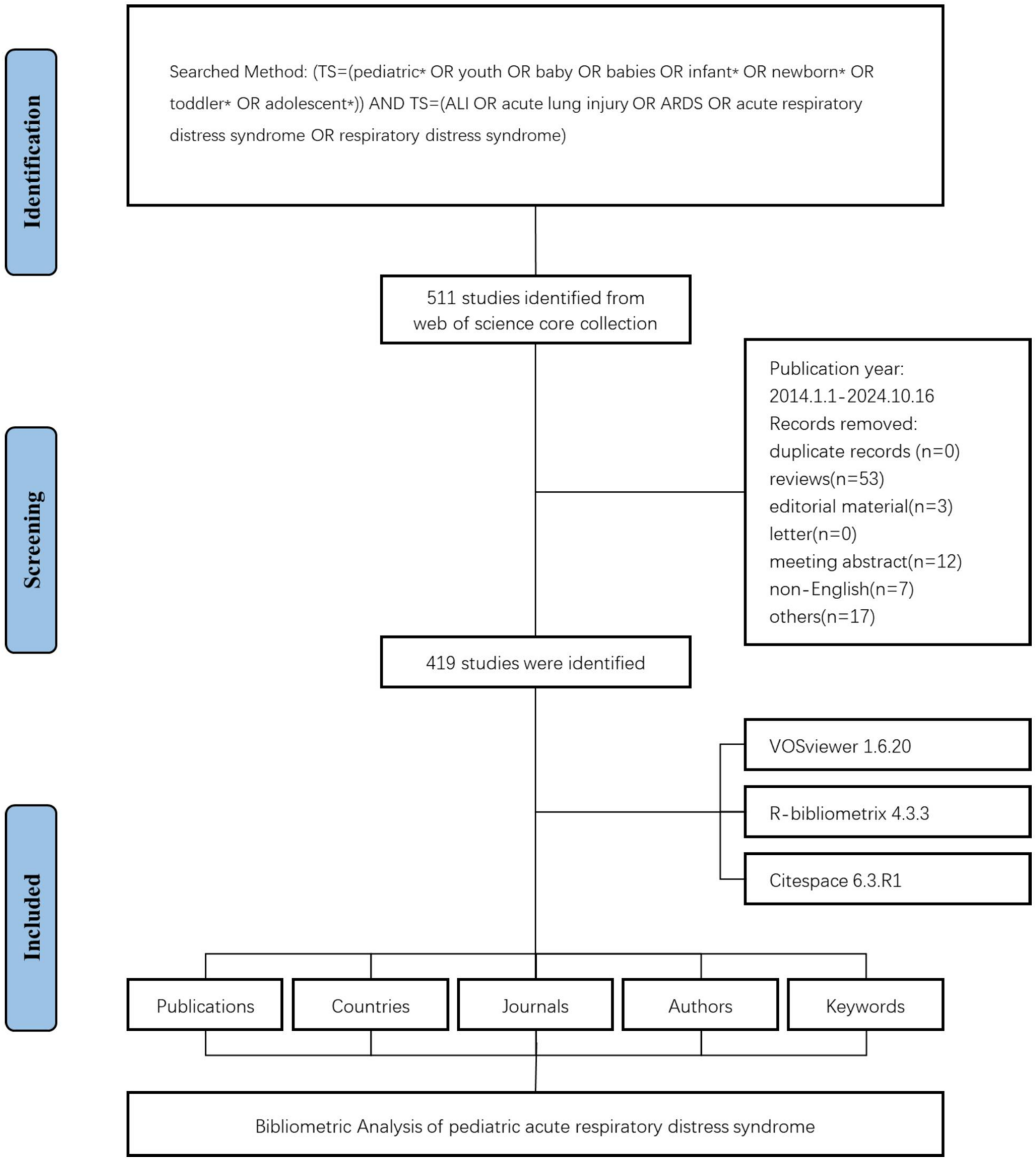
Flowchart of data screening process

The article selection process involved two independent researchers who screened titles and abstracts for relevance to pediatric ARDS/ALI. Any disagreements were resolved through discussion or by consulting a third researcher. After initial screening, the full texts were reviewed to confirm eligibility. The inclusion criteria were: articles published in English, focused on ARDS or ALI in pediatric populations (including neonates, infants, children, and adolescents), and indexed as original research articles or reviews in WoSCC. Exclusion criteria comprised non-English publications, editorials, letters, meeting abstracts, duplicate records, and studies not directly addressing pediatric ARDS/ALI. The selection process and article flow are illustrated in Fig. 1, which details the number of records identified, screened, excluded, and included at each stage. Ultimately, 419 studies were included for bibliometric analysis, following the removal of reviews (*n* = 53), editorial materials (*n* = 3), meeting abstracts (*n* = 12), non-English articles (*n* = 7), and other unrelated records (*n* = 17).

Data were extracted in “Full record and cited references” plain text format, and included publication counts, citation numbers, article titles, author information, institutional affiliations, countries or regions, keywords, and journal names. The methodology for article selection and screening was based on established bibliometric analysis protocols referenced in similar studies. As this study did not involve primary patient data, no patient-specific characteristics were assessed or reported.

### Statistical Analysis

We gathered relevant data from a literature citation database and utilized Microsoft Excel for identifying and calculating bibliometric indicators, covering aspects such as annual publication counts, citation frequencies, average citations, journal names, impact factors (IFs), publishing countries/regions, institutions, and authors. Excel facilitated the effective organization and analysis of this bibliometric data. For visual analysis, we employed three robust bibliometric analysis tools: VOSviewer 1.6.20, CiteSpace 6.3.R1, and R 4.3.3. VOSviewer is versatile and crucial for visualizing relationships such as institutional collaborations, author affiliations, co-authorships, citations, and co-citations [17]. Using VOSviewer, we explored complex connections within collaboration networks and academic domains, providing deeper insights into relationships among authors, institutions, and publications. To further grasp emerging trends and research hotspots in our field, we conducted keyword co-occurrence analysis with VOSviewer and keyword occurrence detection using CiteSpace software. CiteSpace 6.1.R3 was utilized for this analysis, with parameters set from January 2014 to October 2024, focusing on the evolving landscape of PARDS through annual time slices. Node types were defined as keywords, with a node threshold (N) set to pre-pruning threshold = 5, and pruning performed using pathfinder + merging networks. Visual analysis generated a timeline highlighting key keywords in this research area. R 4.3.3 was extensively used to conduct statistical analysis and advanced bibliometric computations, which was also allowed for sophisticated data manipulation, trend analysis, and network analysis.

The size of nodes indicates the number of publications, the thickness of lines reflects the strength of the link, and node color signifies different clusters or periods. We utilized the H-index to measure the academic impact of both individuals and journals. The H-index is a crucial metric for assessing researchers’ academic contributions and predicting their future scientific achievements [18, 19]. In this study, we obtained the H-index for each author from WoSCC. Complementing this, the G-index was utilized to emphasize the broader citation performance across a scholar’s publication portfolio, while the M-index provided an annualized measure of productivity by adjusting the H-index relative to the duration of the researcher’s active career. Further, we extended our analysis to include journal-specific metrics such as the IF and Journal Citation Reports (JCR) rankings for the year 2023. The IF, calculated as the average number of citations received per paper published in the journal over the previous two years, alongside the JCR quartile rankings, which classify journals based on their relative influence within specific subject categories, were instrumental in evaluating the stature and dissemination influence of the publications identified in our study. These indices were sourced from the WoSCC database, providing a robust framework for evaluating both individual and collective academic contributions within the field of pediatric acute respiratory distress syndrome (PARDS).

## Results

### Overview of Publications in Pediatric Acute Respiratory Distress Syndrome Research

**Fig. 2:**
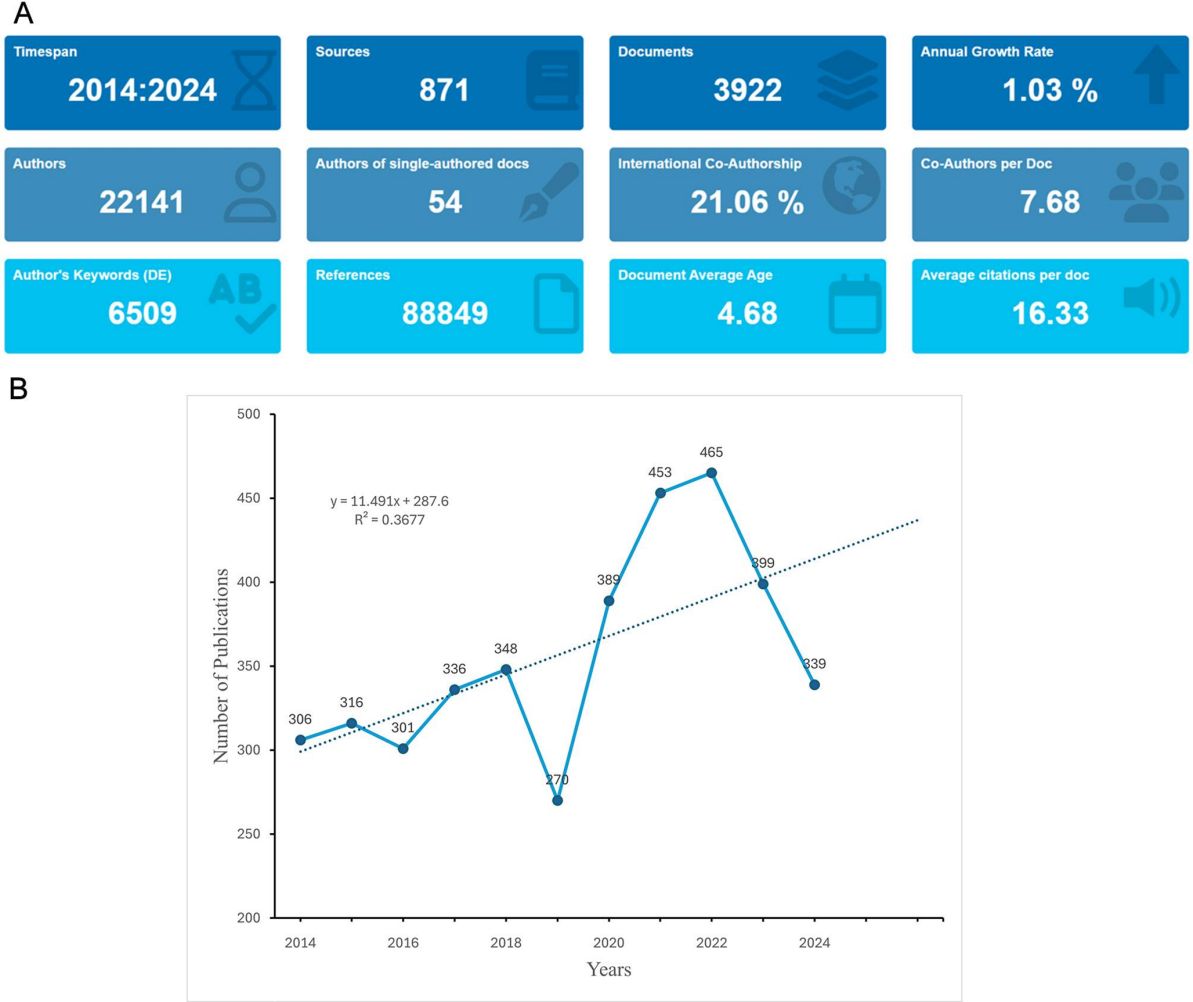
Overview and trends in publications on acute respiratory distress syndrome. (**A**) Number of publications on acupuncture therapy for acute respiratory distress syndrome from 1981 to 2024. (**B**) Annual number of publications on acute respiratory distress syndrome from 1981 to 2024

The data selection process employed in this study was illustrated in Fig. 1. In summary, this study examined 419 publications related to children with ARDS from 2014 to 2024. Our investigation revealed contributions from 22,141 authors, including 54 single-authored documents (Fig. 2A). These authors were affiliated with institutions worldwide, reflecting a global collaboration. The international co-authorship rate was 21.06%, with an average of 7.68 co-authors per document. The analyzed works were published in 871 journals and cited a total of 88,849 references. The annual growth rate of publications in this field is 1.03%, indicating increasing research interest. The documents have an average age of 4.68 years and each received an average of 16.33 citations, highlighting their significant impact and relevance within the scientific community. Additionally, authors used 6,509 unique keywords to describe their research topics, indicating a diverse range of focus areas within PARDS research.

The most cited article closely related to pediatric acute respiratory distress syndrome (PARDS) is “European Consensus Guidelines on the Management of Respiratory Distress Syndrome − 2019 Update” published in *Neonatology* (IF = 2.6) in 2019, which has accumulated a total of 691 citations [20]. The second most cited article is “Surviving Sepsis Campaign International Guidelines for the Management of Septic Shock and Sepsis-Associated Organ Dysfunction in Children” published in *Pediatric Critical Care Medicine* (IF = 4.0) in 2020, with 659 citations [21]. The third most cited article is titled “Recommendations for mechanical ventilation of critically ill children from the Pediatric Mechanical Ventilation Consensus Conference (PEMVECC)” also published in *Pediatric Critical Care Medicine* in 2015, which has received 617 citations [22]. These highly cited articles highlight key advancements and pivotal findings in PARDS, reflecting their importance and influence on subsequent research in the field.

The number of publications related to PARDS has shown a fluctuating yet overall increasing trend over the years (Fig. 2B). Starting from 306 publications in 2014, the field experienced modest growth, with the number of publications reaching 336 in 2017. A notable dip occurred in 2019, where the publication count dropped to 270. However, this was followed by a rapid recovery, with publications peaking at 465 in 2021. The subsequent years saw a slight decline, with 399 publications in 2022 and 339 in 2024. Despite these fluctuations, the linear trend line suggests a long-term upward trend in research output, reflecting the growing recognition of the importance of PARDS and the increasing efforts to address this critical condition. The estimated annual growth rate, based on the trendline, is approximately 11.49 publications per year, further highlighting the expanding research interest in the field.

### Exploration of Journal Impact and Collaboration Networks

The bibliometric analysis identified several high-impact journals contributing significantly to the field of PARDS. Key bibliometric indicators such as the H-index, total publications (TP), total citations (TC), and impact factors (IF) were evaluated to assess the influence and productivity of these journals. The top 20 journals in terms of publication volume published a total of 741 papers (Supplementary Table 1). *Pediatric Critical Care Medicine* stands out as the most prolific journal with 159 total publications and an H-index of 31. *Critical Care Medicine* remains highly influential, with a total citation count of 3,377, ranking fourth in terms of citations. Despite its lower publication count (64 papers), its H-index of 26 reflects its significant impact on the field. *Pediatrics* ranks first in total citations (6,234), highlighting its broad influence, despite a relatively lower publication count of 35. Other notable journals include *Neonatology* and *Pediatric Pulmonology*, which have also contributed substantial research output and influence in this domain.

**Fig. 3:**
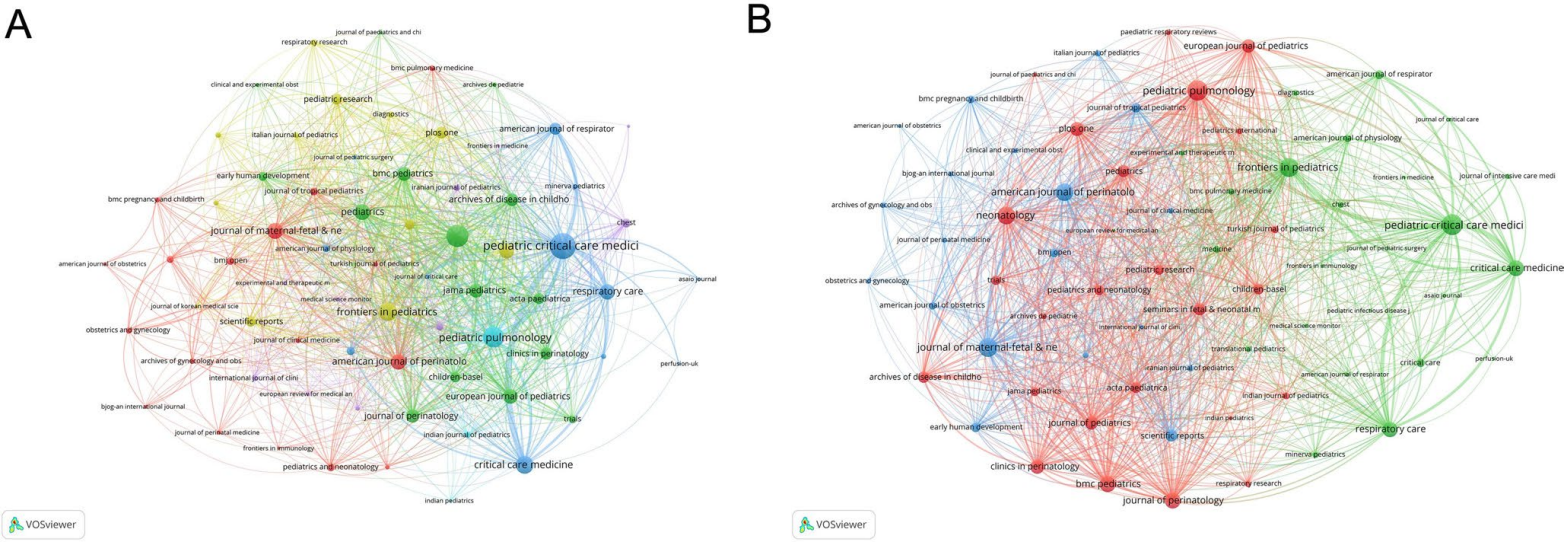
Network analyses of journals in children with acute respiratory distress syndrome. (**A**) The co-occurrence networks of journals. Journal link strength in co-occurrence networks measures the frequency with which two journals are cited together within the same articles or references. (**B**) The coupling networks of journals. Journal link strength in coupling networks assesses the extent to which journals are linked based on the common references cited in their articles

The analysis of journal co-occurrence networks (Fig. 3A) reveals citation relationships among various journals. The co-occurrence network includes 69 journals with at least 10 occurrences. The three key journals with the highest total link strength were *Pediatric Critical Care Medicine* (921), *Neonatology* (650), and *Pediatric Pulmonology* (591). Similarly, the analysis of journal coupling networks (Fig. 3B**)** examines the interconnectedness of journals based on shared references. This network contains 69 journals with at least 10 couples. The three key journals with the highest total link strength were *Pediatric Critical Care Medicine* (41,619), *Pediatric Pulmonology* (39,900), and *Frontiers in Pediatrics* (35,521). The high link strength among these journals highlights their central role in advancing research in PARDS by drawing from a common body of knowledge.

### Analysis of High-Output Authors and their Scientific Collaboration Networks

The top 20 productive authors in PARDS research are listed in Supplementary Table 2. Yehya Nadir has published the most papers (*n* = 61), followed by Thomas Neal J. (*n* = 46) and Khemani Robinder G. (*n* = 44). De Luca Daniele ranks first in total citations with 2,450 and has an H-index of 22, underscoring the significant academic influence of his research. Thomas Neal J. also holds a high H-index of 21, with 1,886 total citations, reflecting both productivity and impact. Other notable authors include Yehya Nadir (H-index 21, 1,419 citations), Khemani Robinder G. (H-index 19, 1,469 citations), and Sapru Anil (H-index 19, 1,382 citations).

**Fig. 4:**
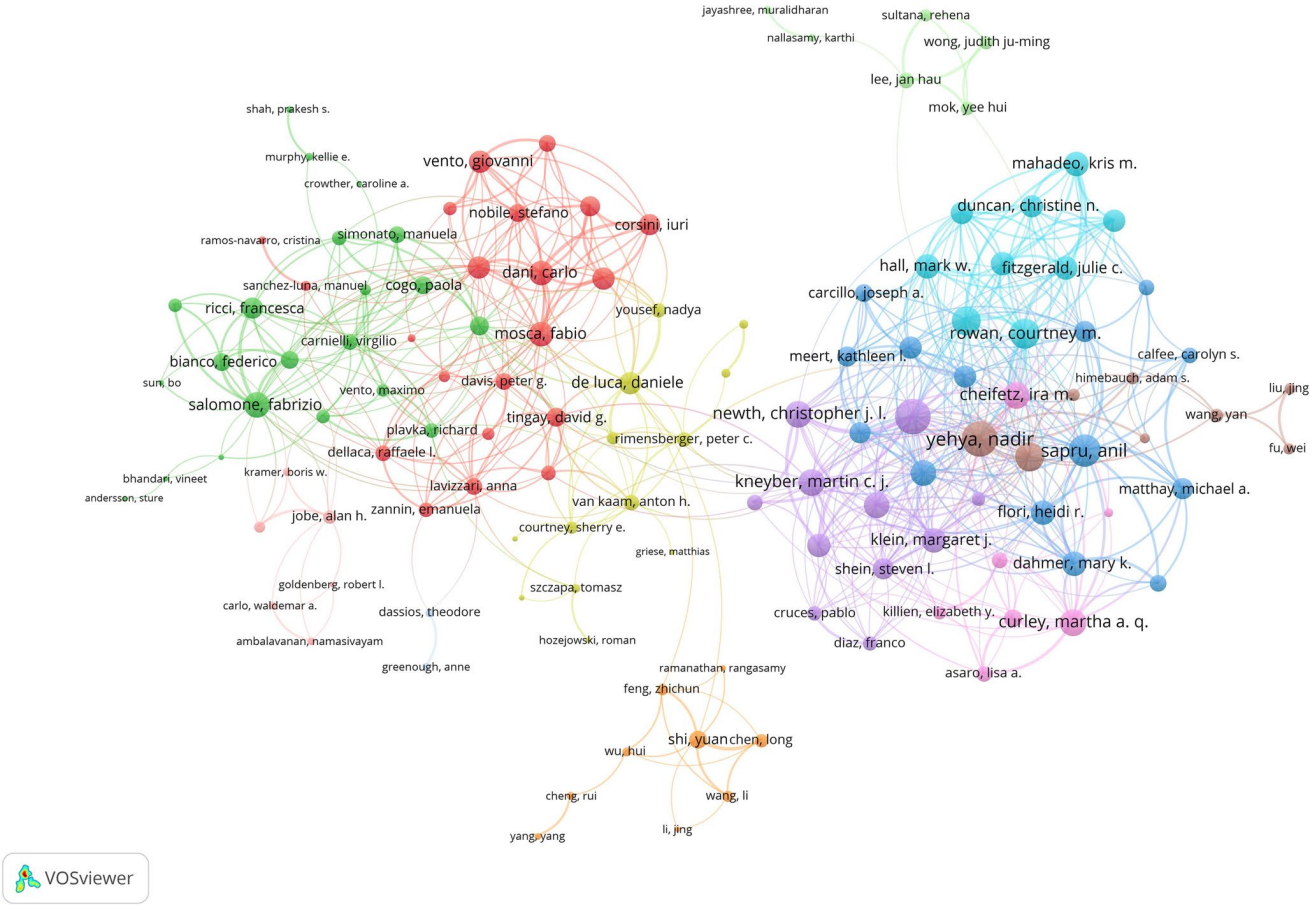
Author collaboration network in children with acute respiratory distress syndrome. Nodes represent authors, with size indicating publication count. Links represent co-authorships, with thickness showing collaboration strength. Colors indicate different research clusters. Total link strength in collaboration networks measures the frequency of co-authorship between authors, indicating the level of collaborative research

A detailed visualization of the co-authorship network among key authors was presented in Fig. 4. The network includes 148 authors with a minimum of 8 articles. Yehya Nadir leads in terms of collaborations with other countries, with 125 total link strength, followed closely by Khemani Robinder G. (124) and Sapru Anil (101). This extensive co-authorship network highlights the collaborative nature of PARDS research, where key authors work together across institutions and countries to advance scientific understanding and improve clinical outcomes. The visualization also reveals clusters of collaboration, with authors like Cheifetz Ira M., Curley Martha A. Q., and Newth Christopher J. L. playing central roles in fostering these collaborative efforts.

### Contributions of Countries and Institutions Trends in PARDS Research

**Fig. 5:**
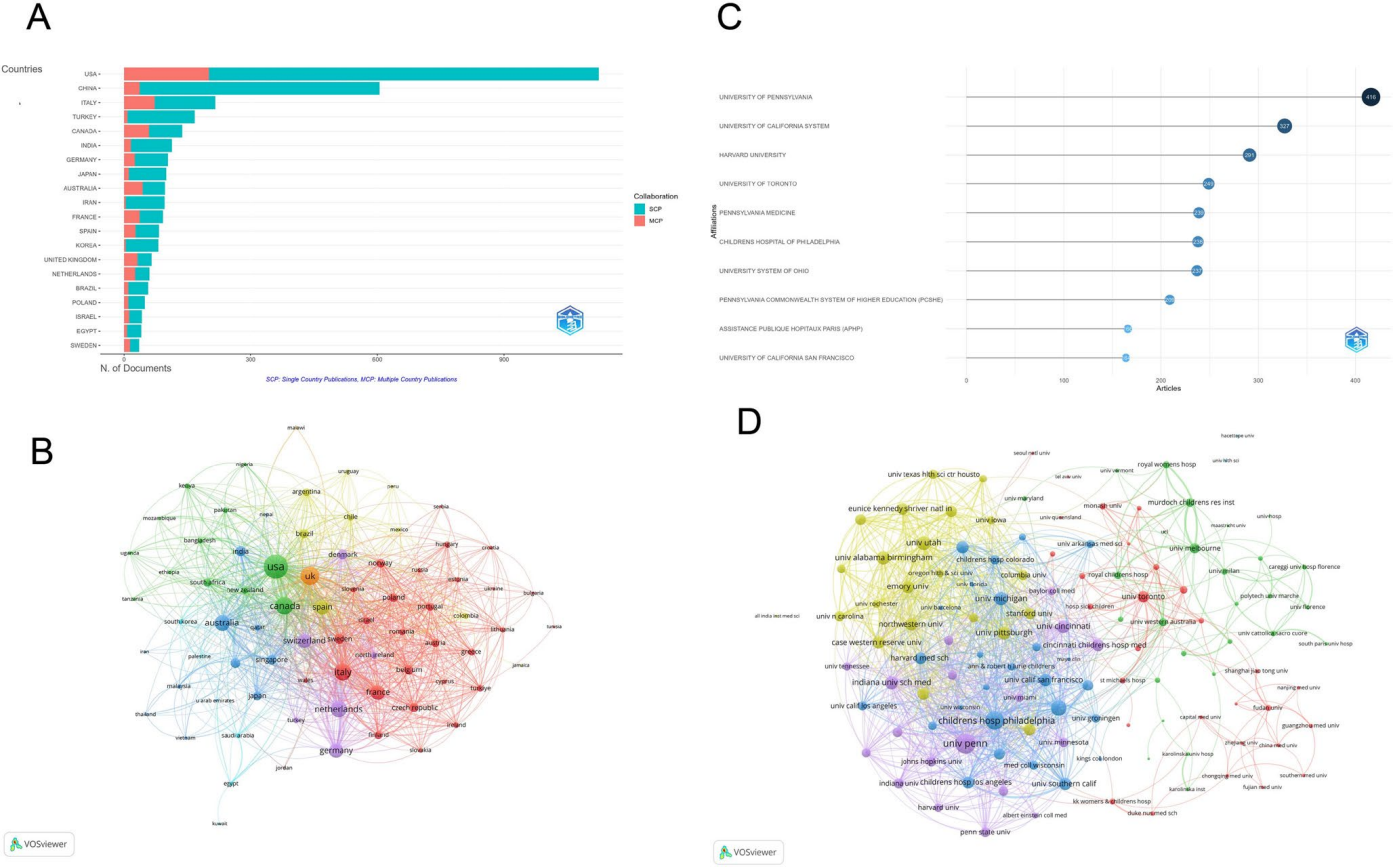
Global distribution and institutional collaboration in children with acute respiratory distress syndrome. (**A**) Distribution of corresponding authors’ publications by Single Country Publications (SCP) and Multiple Country Publications (MCP). (**B**) Visualization map depicting the collaboration among different countries. Countries publication volume and cooperation network diagram: the color depth of the color block represents the size of the publication volume. (**C**) Top 10 institutions by article count and rank. The circle size shows the article count, with darker shades indicating higher ranks. (**D**) Visualization map depicting the collaboration among different institutions. Nodes represent institutions, with size indicating publication count. Links represent co-authorships, with thickness showing collaboration strength. Colors indicate different research clusters. Total link strength in collaboration networks measures the frequency of co-authorship between institutions, indicating the level of collaborative research

The analysis of publication and citation profiles reveals significant contributions from various countries to PARDS research. The top 20 productive countries generated 1,863 articles, accounting for 88.76% of global research output in this field (Fig. 5A and Supplementary Table 3). The United States (USA) remains the most productive country, contributing 1,124 articles, followed by China (*n* = 605) and Italy (*n* = 216). Other notable contributors include Turkey (*n* = 167), Canada (*n* = 138), and India (*n* = 113). The USA’s research impact is further highlighted by its TC count of 24,421 and an average of 21.7 citations per article, making it a leading country both in publication volume and research impact. Canada shows strong performance with a high average citation count of 22.2, while France has the highest average citations per article at 26.9. Countries with high Multiple Country Publication (MCP) ratios—including Australia (MCP ratio = 0.454, total documents = 97), Canada (MCP ratio = 0.428, total documents = 138), and the Netherlands (MCP ratio = 0.433, total documents = 60)—demonstrate robust international collaboration.

The analysis of international collaboration (Fig. 5B) reveals substantial global cooperation in PARDS research, involving 75 countries with a minimum of five published articles. The USA leads with the highest number of collaborations (803 with other countries), followed by the United Kingdom (UK) (483 collaborations) and Canada (441 collaborations). The visualization map shows that the USA, UK, and Canada are central nodes in the global collaboration network, fostering extensive research partnerships across multiple countries. Other key countries in international collaboration include Italy, Netherlands, and Germany, which also exhibit strong link strength.

In bibliometrics, analyzing institutions helps identify research organizations that have made significant contributions to their field and determine their collaborative relationships. The top 10 institutions with the highest research output in PARDS were displayed in Fig. 5C. The University of Pennsylvania leads with 416 articles, highlighting its prominent role in advancing PARDS research. The University of California System follows closely with 327 articles, reflecting its substantial contribution. Harvard University also stands out with 291 publications, underscoring its strong research presence in this field. Other notable institutions include the University of Toronto (249 articles) and the Children’s Hospital of Philadelphia (238 articles), both of which are significant contributors to the research output in PARDS.

A detailed visualization of the collaboration network among institutions involved in PARDS research was provided in Fig. 5D. The University of Pennsylvania emerges as the most collaborative organization, with a total of 496 international collaborations, demonstrating its extensive global network. The Children’s Hospital of Philadelphia also has an impressive collaboration record with 409 international partnerships, further emphasizing its role in fostering research cooperation. The University of Washington (281 collaborations) and University of Michigan (243 collaborations) are also key players in the collaborative landscape, highlighting their active involvement in global research efforts.

**Keyword Co-occurrence Network AND Keyword Bursts: Revealing the Core Focus of PARDS Research**.

**Fig. 6:**
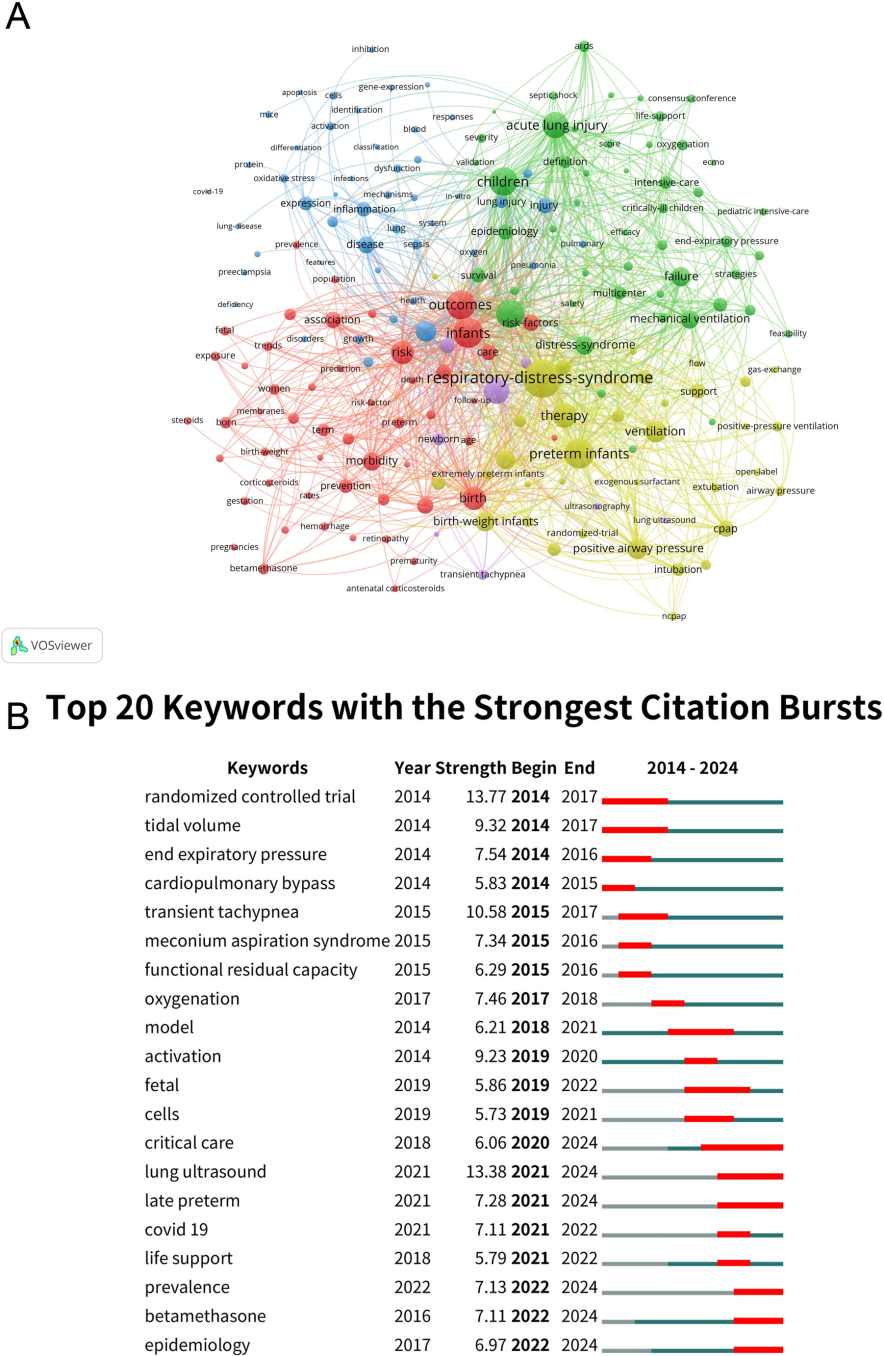
Keyword co-occurrence network and burst analysis of keywords in children with acute respiratory distress syndrome. (**A**) This network visualization displays the co-occurrence of keywords in selected literature. Each node represents a keyword, with size indicating its frequency of occurrence. Links between nodes represent co-occurrence in the same documents, with thicker lines showing stronger associations. Colors reflect the average publication year of the articles, as indicated by the color gradient at the bottom right. (**B**) Top 20 keywords with the strongest citation bursts from 2014 to 2024. The green lines represent the period, and the red lines indicate the burst periods of the keywords

The keyword co-occurrence network analysis reveals five distinct clusters that highlight the main research themes in PARDS **(**Fig. 6A**)**.

### Cluster 1: Neonatal and Infant Outcomes (Red Cluster)

This cluster focuses on neonatal populations, with keywords like “infants” (510 occurrences, 1,762 link strength), “preterm infants” (419 occurrences, 1,780 link strength), and “low-birth-weight”. The emphasis is on risk factors such as “mortality” (360 occurrences, 1,612 link strength) and “morbidity” (139 occurrences, 682 link strength), reflecting the study of outcomes in preterm and low-birth-weight infants.

### Cluster 2: Acute Respiratory Distress and Critical Care (Green Cluster)

This cluster centers on “respiratory distress syndrome” (751 occurrences, 2,873 link strength) and the management of “ARDS” and “acute lung injury” (354 occurrences, 1,373 link strength). Terms such as “mechanical ventilation” (155 occurrences, 686 link strength) and “ECMO” emphasize critical care and life-support strategies for severe respiratory failure.

### Cluster 3: Inflammation and Disease Mechanisms (Blue Cluster)

The focus here is on disease mechanisms, with terms like “inflammation” and “oxidative stress”, reflecting ongoing research into the molecular pathways of lung injury. This cluster also includes “infection” and “sepsis”, highlighting the role of immune responses in respiratory distress.

### Cluster 4: Respiratory Support and Interventions (Yellow Cluster)

This cluster emphasizes respiratory support strategies, including “ventilation” (200 occurrences, 929 link strength) and “positive airway pressure” (175 occurrences, 811 link strength). It also includes terms related to clinical interventions like “CPAP” and “exogenous surfactant”, crucial for managing neonatal respiratory distress.

### Cluster 5: Diagnostic Tools and Guidelines (Purple Cluster)

Keywords like “lung ultrasound”, “diagnosis”, and “guidelines” dominate this cluster, showcasing the importance of diagnostic tools and management protocols in treating PARDS.

Bibliometrics identifies hotspots and trends in the research field by analyzing many academic literatures. Through the keyword burst analysis shown in Fig. 6B, the most notable burst is for “randomized controlled trial” (2014–2017, strength 13.77), underscoring the role of clinical trials in shaping treatment protocols. Other terms related to ventilation, such as “tidal volume” (2014–2017, strength 9.32) and “end expiratory pressure” (2014–2016, strength 7.54), highlight the early focus on optimizing respiratory support strategies. Additional bursts in keywords like “cardiopulmonary bypass” (2014–2015, strength 5.83) and “transient tachypnea” (2015–2017, strength 10.58) reflect research on advanced life support techniques and neonatal respiratory conditions, respectively. Emerging interest in “meconium aspiration syndrome” (2015–2016, strength 7.34) and “functional residual capacity” (2015–2016, strength 6.29) indicates attention to neonatal lung function and related complications.

From 2017 onwards, terms like “oxygenation” (2017–2018, strength 7.46) and “model” (2018–2021, strength 6.21) show a growing focus on improving oxygenation approaches and modeling disease mechanisms. The burst of “activation” (2019–2020, strength 9.23) suggests an interest in inflammatory and cellular responses during this period. More recent bursts reflect the evolving landscape of PARDS research. For instance, “critical care” (2020–2024, strength 6.06) and “lung ultrasound” (2021–2024, strength 13.38) highlight the increased focus on critical care strategies and non-invasive diagnostic tools. Additionally, “late preterm” (2021–2024, strength 7.28) and “covid 19” (2021–2022, strength 7.11) illustrate the impact of neonatal prematurity and the pandemic on respiratory research. Emerging trends are also evident in keywords like “prevalence” (2022–2024, strength 7.13), “betamethasone” (2022–2024, strength 7.11), and “epidemiology” (2022–2024, strength 6.97), reflecting a growing interest in population health, steroid use in preterm infants, and epidemiological studies.

## Discussion

The analysis highlights major trends in research on PARDS. From 2014 to 2024, the number of publications on PARDS has shown a fluctuating yet overall increasing trend, with notable growth between 2014 and 2021, peaking at 465 publications. Despite a slight decrease in the following years, the overall upward trend reflects increasing research interest in PARDS. The most influential journals in this area were *Pediatric Critical Care Medicine* and *Critical Care Medicine*, which published a substantial number of high-impact studies. The USA remains the most prolific country, contributing the highest number of articles (1,124) and accounting for a significant portion of global research output, with a total of 24,421 citations. Prominent authors, such as Yehya N, Thomas NJ, and Khemani RG, have made significant contributions to the field, with high citation counts and influential research, further shaping the research landscape. Leading institutions, including the University of Pennsylvania, the University of California System, and the Children’s Hospital of Philadelphia, have demonstrated extensive collaborative efforts and have made significant contributions to PARDS research, solidifying their positions at the forefront of this field.

The prominence of *Pediatric Critical Care Medicine* and *Critical Care Medicine* in PARDS research reflects their pivotal role in disseminating high-impact clinical studies and shaping the direction of care standards for this condition. The strong presence of these journals, particularly *Pediatric Critical Care Medicine*, which focuses specifically on the pediatric population, highlights the growing recognition of the unique challenges that PARDS presents in children [23], This journal’s significant output suggests that the pediatric critical care community increasingly acknowledges the need for specialized research and evidence-based treatment strategies tailored to younger patients. On the other hand, *Critical Care Medicine* continues to provide a broader platform for critical care topics, yet its substantial citation impact in the PARDS domain underscores its relevance to both pediatric and adult populations, particularly in the context of shared clinical concerns such as mechanical ventilation and respiratory failure management [24]. The consistent presence of these journals in the PARDS research landscape illustrates their role not only in advancing scientific understanding but also in guiding clinical practices and setting benchmarks for future research directions.

This bibliometric analysis underscores the dominant contributions of the USA, China, and Italy, which have played pivotal roles in advancing PARDS research. The USA, with the highest number of publications and citations, continues to lead the global research landscape, benefiting from its well-established biomedical research infrastructure, significant funding, and a strong network of collaborative institutions. American institutions, such as the University of Pennsylvania and the University of California System, have been instrumental in driving research forward, reflecting the country’s emphasis on innovation and clinical application. China’s rapid rise in PARDS research output is noteworthy, driven by substantial investments in science and technology, as well as growing international collaborations. This reflects China’s increasing focus on pediatric critical care research as its healthcare system evolves and expands. Italy, while contributing a smaller volume of publications compared to the USA and China, has maintained a strong presence in high-quality research, demonstrating the importance of European collaboration and healthcare-driven research initiatives. These countries exemplify how national investments in healthcare and research infrastructure, along with international cooperation, are crucial for advancing scientific understanding and improving clinical outcomes in PARDS. The global nature of PARDS research, as evidenced by the extensive international co-authorship and collaboration networks, highlights the collective effort required to address this complex condition, with each nation’s contributions helping to refine treatment protocols and therapeutic strategies.

The authors highlighted in this analysis, Yehya N, Khemani RG, and Thomas N, have made substantial contributions to the field of pediatric acute respiratory distress syndrome (PARDS) [25]. Their research, reflected in numerous publications, has centered on vital areas like mechanical ventilation, acute lung injury, and extracorporeal membrane oxygenation (ECMO) [26]. These studies have not just enhanced our comprehension of PARDS pathophysiology, but they have also led to significant advancements in clinical practices, thereby improving patient outcomes. Their influence extends beyond their specific research; these authors have played a pivotal role in shaping research trends and directions in the field of pediatric critical care. Their work has been frequently referenced in the literature, serving as a benchmark for subsequent research projects. The impact and visibility of their contributions are further underscored by their publications in highly regarded journals like *Pediatric Critical Care Medicine* and *Critical Care Medicine* [5]. Through their collaborations and partnerships, they have fostered a more holistic approach to PARDS research, bringing together experts from diverse backgrounds. In summary, these authors’ research, collaborations, and impact on clinical practice have significantly influenced the current landscape of PARDS management, paving the way for continued progress in this critical area.

### Cluster 1: Neonatal and Infant Outcomes (Red Cluster)

This cluster, with a strong focus on neonatal populations, reflects the ongoing concern with outcomes in vulnerable groups such as preterm and low-birth-weight infants. The prominence of keywords like “preterm infants” and “low-birth-weight” indicates that research has heavily centered on understanding the unique challenges faced by these populations, who are at high risk for respiratory distress [27]. The high occurrence of terms like “mortality” and “morbidity” further underscores the critical need to improve survival rates while minimizing long-term health complications in these infants.

What this cluster reveals are a sustained focus on outcome-driven research, particularly on identifying risk factors and optimizing interventions that can reduce mortality and morbidity [28]. Given the vulnerability of this patient group, future research will likely continue exploring interventions like exogenous surfactant administration and non-invasive ventilation techniques, potentially expanding into more personalized approaches for neonatal care. We can also expect more longitudinal studies that track the long-term outcomes of these infants into childhood and beyond, providing insights into how early interventions impact later life stages.

### Cluster 2: Acute Respiratory Distress and Critical Care (Green Cluster)

This cluster underscores the central role of ARDS and its management in pediatric care. The keywords “respiratory distress syndrome,” “acute lung injury,” and “mechanical ventilation” highlight that the management of severe respiratory failure remains a cornerstone of PARDS research. The inclusion of “ECMO” (extracorporeal membrane oxygenation) points to the growing use of advanced life-support technologies for patients who do not respond to conventional treatments.

The critical-care focus of this cluster suggests that ventilation strategies and life-support technologies will remain a priority in future research. Given the risks associated with mechanical ventilation, such as ventilator-induced lung injury (VILI), ongoing research will likely continue to refine ventilation protocols, particularly in pediatric patients with more fragile lungs [29]. Additionally, as ECMO becomes more accessible, research will need to focus on optimizing patient selection criteria, minimizing complications, and improving long-term outcomes for children who require this invasive intervention [30].

### Cluster 3: Inflammation and Disease Mechanisms (Blue Cluster)

This cluster, featuring terms like “inflammation,” “oxidative stress,” and “sepsis,” points to a growing interest in the molecular and immunological mechanisms underlying ARDS. Inflammation is a well-known driver of lung injury in ARDS, with immune responses often exacerbating the severity of respiratory failure [31]. The inclusion of terms such as “infection” and “sepsis” highlights the importance of immune dysregulation, particularly in cases where ARDS is triggered by infectious agents like bacteria or viruses.

The focus on disease mechanisms suggests that future research will likely delve deeper into the molecular pathways that contribute to lung injury in pediatric ARDS. There is growing interest in identifying key biomarkers that can help predict which patients are at risk for severe inflammatory responses, such as cytokine storms [31]. This could lead to targeted therapies aimed at modulating the immune response, reducing inflammation, and potentially preventing the progression of ARDS in high-risk patients [32]. Additionally, research into the role of oxidative stress could open new avenues for therapeutic interventions that protect the lungs from damage at the cellular level.

### Cluster 4: Respiratory Support and Interventions (Yellow Cluster)

This cluster emphasizes respiratory support techniques, with keywords like “ventilation,” “positive airway pressure,” and “CPAP” (continuous positive airway pressure). These terms reflect a continued focus on optimizing non-invasive and invasive respiratory support strategies, particularly for neonatal and pediatric populations. The inclusion of “exogenous surfactant” also highlights its importance as a therapeutic intervention for neonatal respiratory distress.

The emphasis on these support strategies suggests that research will continue focusing on improving non-invasive ventilation methods to avoid the complications associated with mechanical ventilation [33]. Given the fragility of neonatal lungs, interventions like CPAP and surfactant replacement therapy have become crucial in managing early-stage respiratory distress [34]. Future studies might explore how to personalize these interventions further, adjusting them to the specific needs of different subpopulations. For instance, research could investigate how CPAP settings or surfactant dosages might be optimized for infants based on gestational age, birth weight, or underlying health conditions.

### Cluster 5: Diagnostic Tools and Guidelines (Purple Cluster)

The fifth cluster, dominated by terms like “lung ultrasound” and “diagnosis,” highlights the role of diagnostic tools and clinical guidelines in managing PARDS. The growing use of lung ultrasound as a non-invasive, bedside diagnostic tool reflects a shift towards more accessible and less harmful methods of assessing lung pathology in pediatric patients. This cluster also suggests that the development of standardized guidelines is becoming increasingly important for ensuring consistent, high-quality care across different clinical settings.

The focus on diagnostic tools indicates that future research will likely prioritize the validation and standardization of non-invasive diagnostic methods, such as lung ultrasound. As more pediatric intensive care units (PICUs) adopt this technology, research will need to focus on refining its diagnostic accuracy and expanding its applications [35]. Furthermore, the development and dissemination of evidence-based guidelines will be critical for ensuring that best practices are followed in diagnosing and managing ARDS [36]. These guidelines will likely incorporate the latest advances in imaging, biomarker detection, and clinical protocols to enhance early diagnosis and improve patient outcomes.

The analysis of keyword bursts from 2014 to 2024 reveals important shifts in research priorities within the field of ARDS. Early bursts (2014–2017) are associated with keywords such as “randomized controlled trial,” “tidal volume, " and “end expiratory pressure*”*. This period reflects a strong focus on clinical trials and the optimization of mechanical ventilation strategies, particularly lung-protective ventilation techniques [37]. These keywords indicate that the primary concern during this time was refining ventilation settings to minimize VILI, a significant risk in pediatric patients with ARDS. Research in this period was instrumental in establishing low tidal volume ventilation as a standard of care for mitigating lung injury in severe cases [38].

As research progressed from 2015 to 2017, keywords such as “transient tachypnea” and “meconium aspiration syndrome” emerged, highlighting the increased focus on neonatal respiratory conditions. These bursts suggest growing attention towards understanding and managing respiratory distress in neonates, which often presents similarly to ARDS but with distinct pathophysiological mechanisms [39]. The inclusion of these neonatal topics in the broader PARDS research reflects the ongoing efforts to tailor treatments based on age-specific vulnerabilities and developmental differences in lung physiology.

Moving into 2018 and beyond, keywords like “model” (2018–2021) and “critical care” (2021–2024) gained prominence, signaling a shift towards the development of predictive models and comprehensive critical care management strategies for PARDS. The burst of “model” suggests that researchers began to focus on creating predictive models for patient outcomes, aiming to better stratify patients based on risk and to tailor interventions accordingly [40]. This aligns with the growing interest in precision and personalized medicine, which seeks to individualize treatment approaches based on predictive biomarkers and clinical variables [41].

In recent years (2020–2024), keywords such as “lung ultrasound,” “life support,” “COVID-19,” and *critical care* have demonstrated strong citation bursts. The rise of “lung ultrasound” (2021–2024) underscores the growing use of point-of-care imaging to diagnose and monitor lung conditions in pediatric patients, offering a non-invasive and readily accessible tool that has gained traction in critical care settings [42]. The burst of “COVID-19” (2021–2022) reflects the global impact of the pandemic on respiratory research, with a particular focus on how SARS-CoV-2 affects lung function in pediatric patients and its role in exacerbating respiratory distress syndromes like ARDS [43]. Interestingly, the emergence of “prevalence and epidemiolog*y*” (2022–2024) as keywords suggests a growing emphasis on understanding the epidemiological aspects of PARDS. This shift may indicate a heightened interest in studying the incidence, risk factors, and outcomes of ARDS in different pediatric populations, potentially driven by the lessons learned from the COVID-19 pandemic. The increased focus on “life support” (2018–2024) likely reflects advances in ECMO and other supportive technologies that are critical for managing severe cases of PARDS. ECMO has become a key research area, with efforts focused on optimizing patient selection, improving outcomes, and reducing long-term complications.

Looking ahead, based on the most recent bursts, future research is likely to concentrate on the application of advanced imaging techniques (lung ultrasound), the long-term impacts of COVID-19 on pediatric respiratory health, and further exploration of *prevalence* and *epidemiology* to better understand the burden of ARDS in children. Additionally, the prominence of *life support* and the ongoing interest in *critical care* suggest that research will continue to focus on improving the management of severe cases through technological innovations and enhanced critical care protocols.

### Strengths and Limitations

A major strength of this study is the use of a bibliometric approach, which enables a comprehensive and quantitative overview of research trends, influential publications, and collaborative networks in PARDS over the past decade. Bibliometric analysis provides objective insights into publication patterns, citation impact, and the evolution of research topics, which are valuable for mapping the development of the field and identifying key contributors and research hotspots. Furthermore, the use of multiple analytical tools (VOSviewer, CiteSpace, and R-bibliometrix) enhances the robustness and depth of the analysis by enabling visualization and cross-validation of bibliometric networks.

However, several limitations inherent to bibliometric methods should be considered. First, this analysis relies solely on the WoSCC database, which, while comprehensive, may not include all relevant publications indexed in other databases such as Scopus or PubMed, potentially leading to selection bias. Second, bibliometric indicators such as citation counts may not fully reflect the true clinical impact or quality of individual articles, as highly cited papers are not always the most clinically relevant. Third, only articles published in English were included, which may lead to language bias and underrepresentation of research from non-English-speaking regions. Additionally, the analysis is subject to database updates and changes in citation practices over time. Lastly, author and institution name variations may affect the accuracy of collaboration and productivity assessments. Despite these limitations, bibliometric analysis remains a powerful tool for gaining a macro-level perspective of research activity and guiding future studies in PARDS.

## Conclusion

This bibliometric analysis provides a comprehensive overview of global research trends in PARDS over the past decade. The study demonstrates a marked increase in research activity, with key thematic focuses on mechanical ventilation, acute lung injury, extracorporeal membrane oxygenation, and the development of diagnostic tools such as lung ultrasound. While leading countries, institutions, and authors have significantly shaped the field, our analysis also reveals important research trends, including the growing emphasis on personalized critical care strategies, inflammation and molecular mechanisms, and advances in respiratory support technologies. Despite this progress, notable research gaps remain. There is a need for greater diversity in study populations, more long-term outcome studies, and further investigation into cellular mechanisms and biomarker-driven approaches. Additionally, the standardization of diagnostic criteria and treatment protocols remains an ongoing challenge. By identifying these trends and gaps, this study provides valuable direction for future research aimed at improving the diagnosis, management, and outcomes of children with PARDS. Continued bibliometric analyses will be essential for monitoring advances in the field, identifying emerging areas of interest, and guiding collaborative efforts to address unmet clinical needs.

## Electronic Supplementary Material

Below is the link to the electronic supplementary material.

Supplementary Material 1

## Data Availability

The datases supporting the conclusions of this article are included within the article and its additional files.

## Abbreviations

ARDS: Acute respiratory distress syndrome
PARDS: Pediatric ARDS
WoSCC: Web of Science Core Collection
ECMO: Extracorporeal membrane oxygenation
PICUs: Pediatric intensive care units
USA: United States
MCP: Multiple Country Publication
VILI: Ventilator-induced lung injury
