# Supplementary material for: Global Research Trends in Pediatric Acute Respiratory Distress Syndrome: A Bibliometric Analysis from 2014 To 2024
Source: J Epidemiol Glob Health. 2025 Jun 20;15(1):87. doi: 10.1007/s44197-025-00434-6 (PMC12181164; doi:10.1007/s44197-025-00434-6)
Supplement: Supplementary file 1 — Supplementary Material 1 [file 44197_2025_434_MOESM1_ESM.docx]

Supplementary Table 1. Bibliometric Indicators of High-Impact Journals.

| **Journal** | **H_index** | **IF (2023)** | **JCR_Quartile (2023)** | **PY_start** | **TP** | **TP_rank** | **TC** | **TC_rank** |
| --- | --- | --- | --- | --- | --- | --- | --- | --- |
| Pediatric Critical Care Medicine | 31 | 4 | Q1 | 2014 | 159 | 1 | 3153 | 6 |
| Critical Care Medicine | 26 | 7.7 | Q1 | 2014 | 64 | 10 | 3377 | 4 |
| Pediatrics | 25 | 6.2 | Q1 | 2014 | 35 | 18 | 6234 | 1 |
| Neonatology | 23 | 2.6 | Q1 | 2014 | 68 | 9 | 2156 | 11 |
| American Journal of Obstetrics and Gynecology | 22 | 8.7 | Q1 | 2014 | 31 | 20 | 2450 | 7 |
| Journal of Maternal-Fetal & Neonatal Medicine | 21 | 1.7 | Q3 | 2014 | 144 | 2 | 1248 | 21 |
| Pediatric Pulmonology | 21 | 2.7 | Q2 | 2014 | 122 | 4 | 1811 | 16 |
| PLOS One | 21 | 2.9 | Q1 | 2014 | 88 | 6 | 1466 | 17 |
| Journal of Pediatrics | 20 | 3.9 | Q1 | 2014 | 49 | 12 | 3459 | 3 |
| BMC Pediatrics | 19 | 2 | Q2 | 2014 | 70 | 7 | 606 | 33 |
| Journal of Perinatology | 17 | 2.4 | Q1 | 2014 | 60 | 11 | 1971 | 12 |
| Pediatric Research | 17 | 3.1 | Q1 | 2014 | 48 | 13 | 2235 | 9 |
| Respiratory Care | 17 | 2.4 | Q2 | 2014 | 70 | 8 | 707 | 29 |
| American Journal of Perinatology | 16 | 1.5 | Q2 | 2014 | 114 | 5 | 989 | 24 |
| European Journal of Pediatrics | 16 | 3 | Q1 | 2014 | 46 | 14 | 953 | 25 |
| Archives Of Disease in Childhood-Fetal And Neonatal Edition | 15 | 3.9 | Q1 | 2014 | 27 | 23 | 2372 | 8 |
| Jama Pediatrics | 15 | 24.7 | Q1 | 2014 | 17 | 40 | 891 | 26 |
| American Journal of Physiology-Lung Cellular And Molecular Physiology | 14 | 3.6 | Q1 | 2014 | 31 | 21 | 1403 | 19 |
| American Journal of Respiratory And Critical Care Medicine | 14 | 19.3 | Q1 | 2014 | 15 | 44 | 3324 | 5 |
| American Journal of Respiratory Cell And Molecular Biology | 14 | 5.9 | Q1 | 2014 | 15 | 45 | 648 | 32 |

H_index: Measures both the productivity and citation impact of the publications in the journal. IF (Impact Factor): Indicates the average number of citations to recent articles published in the journal. JCR_Quartile: Ranks the journal within its field according to the Journal Citation Reports, with quartiles indicating the position (Q1: top 25%, Q2: 25%-50%, Q3: 50%-75%, Q4: bottom 25%). TP (Total Publications): Total number of publications by the journal. TP_rank: Rank of the journal based on the total number of publications. TC (Total Citations): Total number of citations received by the journal's articles. TC_rank: Rank of the journal based on the total number of citations. PY_start (Publication Year Start): The year when the journal first started publishing.

Supplementary Table 2. Publication and Citation Profiles of High-Impact Authors.

| **Authors** | **H_index** | **g-index** | **m-index** | **PY_start** | **TP** | **TP_Frac** | **TP_rank** | **TC** | **TC_rank** |
| --- | --- | --- | --- | --- | --- | --- | --- | --- | --- |
| De Luca Daniele | 22 | 38 | 2.00 | 2014 | 38 | 6.04 | 4 | 2450 | 1 |
| Thomas Neal J. | 21 | 43 | 1.91 | 2014 | 46 | 8.06 | 2 | 1886 | 4 |
| Yehya Nadir | 21 | 36 | 1.91 | 2014 | 61 | 11.11 | 1 | 1419 | 7 |
| Khemani Robinder G. | 19 | 38 | 1.73 | 2014 | 44 | 6.05 | 3 | 1469 | 6 |
| Sapru Anil | 19 | 33 | 1.90 | 2015 | 33 | 3.44 | 5 | 1382 | 9 |
| Curley Martha A. Q. | 17 | 32 | 1.70 | 2015 | 32 | 4.46 | 6 | 1398 | 8 |
| Newth Christopher J. L. | 17 | 31 | 1.55 | 2014 | 31 | 4.32 | 8 | 1732 | 5 |
| Cheifetz Ira M. | 16 | 27 | 1.46 | 2014 | 27 | 4.09 | 11 | 2131 | 2 |
| Kneyber Martin C. J. | 16 | 28 | 1.46 | 2014 | 28 | 4.35 | 10 | 1971 | 3 |
| Dani Carlo | 14 | 26 | 1.27 | 2014 | 31 | 3.91 | 7 | 714 | 22 |
| Emeriaud Guillaume | 14 | 21 | 1.40 | 2015 | 21 | 2.08 | 18 | 1171 | 14 |
| Mosca Fabio | 14 | 25 | 1.27 | 2014 | 25 | 1.80 | 13 | 732 | 21 |
| Davis Peter G. | 13 | 24 | 1.18 | 2014 | 24 | 3.48 | 15 | 612 | 26 |
| Flori Heidi R. | 13 | 20 | 1.30 | 2015 | 20 | 2.43 | 19 | 1185 | 12 |
| Liu Jing | 13 | 18 | 1.18 | 2014 | 18 | 3.88 | 23 | 654 | 24 |
| Matthay Michael A. | 12 | 15 | 1.20 | 2015 | 15 | 1.54 | 34 | 466 | 29 |
| Shi Yuan | 12 | 21 | 1.09 | 2014 | 30 | 5.13 | 9 | 449 | 30 |
| Watson R. Scott | 12 | 18 | 1.20 | 2015 | 18 | 2.42 | 24 | 1180 | 13 |
| Jouvet Philippe | 11 | 18 | 1.00 | 2014 | 18 | 2.42 | 22 | 1141 | 15 |
| Lista Gianluca | 11 | 20 | 1.00 | 2014 | 22 | 2.05 | 16 | 401 | 34 |

H_index: Measures both the productivity and citation impact of the author's publications. g_index: Gives more weight to highly-cited articles, emphasizing the impact of top-performing papers. m_index: The h-index divided by the number of years since the author's first published paper, measuring consistent impact over time. TP (Total Publications): Total number of publications by the author. TP_rank: Rank of the author based on the total number of publications. TP_FRAC (Fraction of Total Publications): Proportion of the author's publications relative to the total number of publications in the dataset. TC (Total Citations): Total number of citations received by the author's publications. TC_rank: Rank of the author based on the total number of citations. Average Citations: The average number of citations per publication by the author. PY_start (Publication Year Start): The year when the author first started publishing.

Supplementary Table 3. Publication and Citation Profiles of Leading Countries.

| **Country** | **Articles** | **Freq** | **MCP_Ratio** | **TP** | **TP_rank** | **TC** | **TC_rank** | **Average Citations** |
| --- | --- | --- | --- | --- | --- | --- | --- | --- |
| USA | 1124 | 0.287 | 0.179 | 6178 | 1 | 24421 | 1 | 21.7 |
| China | 605 | 0.154 | 0.061 | 2254 | 2 | 8299 | 2 | 13.7 |
| Italy | 216 | 0.055 | 0.333 | 1094 | 3 | 3636 | 3 | 16.8 |
| Turkey | 167 | 0.043 | 0.048 | 434 | 10 | 1404 | 9 | 8.4 |
| Canada | 138 | 0.035 | 0.428 | 870 | 4 | 3061 | 4 | 22.2 |
| India | 113 | 0.029 | 0.142 | 352 | 13 | 1204 | 12 | 10.7 |
| Germany | 104 | 0.027 | 0.240 | 505 | 7 | 1680 | 7 | 16.2 |
| Japan | 100 | 0.025 | 0.110 | 491 | 8 | 867 | 15 | 8.7 |
| Australia | 97 | 0.025 | 0.454 | 619 | 5 | 2233 | 6 | 23 |
| Iran | 96 | 0.024 | 0.042 | 383 | 12 | 600 | 18 | 6.2 |
| France | 92 | 0.023 | 0.402 | 558 | 6 | 2476 | 5 | 26.9 |
| Spain | 83 | 0.021 | 0.325 | 429 | 11 | 1408 | 8 | 17 |
| Korea | 81 | 0.021 | 0.049 | 253 | 16 | 738 | 16 | 9.1 |
| United Kingdom | 65 | 0.017 | 0.492 | 439 | 9 | 1346 | 11 | 20.7 |
| Netherlands | 60 | 0.015 | 0.433 | 316 | 14 | 1146 | 13 | 19.1 |
| Brazil | 57 | 0.015 | 0.175 | 283 | 15 | 363 | 23 | 6.4 |
| Poland | 49 | 0.012 | 0.204 | 205 | 17 | 376 | 22 | 7.7 |
| Israel | 42 | 0.011 | 0.286 | 139 | 20 | 338 | 24 | 8 |
| Egypt | 41 | 0.010 | 0.171 | 129 | 21 | 557 | 19 | 13.6 |
| Sweden | 36 | 0.009 | 0.389 | 178 | 19 | 974 | 14 | 27.1 |

Articles: Publications where the corresponding authors are from a specific country. Freq (Frequency of Total Publications): Frequency of publications from the country relative to the total dataset. MCP_Ratio (Multiple Country Publications Ratio): Proportion of publications involving collaborations with authors from other countries. TP (Total Publications): Total number of publications from the country. TP_rank: Rank of the country based on the total number of publications. TC (Total Citations): Total number of citations received by the country's publications. TC_rank: Rank of the country based on the total number of citations. Average Citations: The average number of citations per publication from the country.
